# Identification of new candidate drugs in myelodysplastic syndromes with splicing factor mutations by transcriptional profiling and connectivity map analysis

**DOI:** 10.1111/bjh.20026

**Published:** 2025-02-23

**Authors:** Tianyu Sun, Shalini Singh, Hayson Chenyu Wang, Juseong Lee, Hamid Dolatshad, Pak Leng Cheong, Douglas R. Higgs, Jacqueline Boultwood, Andrea Pellagatti

**Affiliations:** ^1^ Nuffield Division of Clinical Laboratory Sciences, Radcliffe Department of Medicine University of Oxford Oxford UK; ^2^ Shanghai Ninth People's Hospital Shanghai Jiaotong University School of Medicine Shanghai China; ^3^ Nuffield Department of Surgical Sciences University of Oxford Oxford UK; ^4^ MRC Weatherall Institute of Molecular Medicine, Radcliffe Department of Medicine University of Oxford Oxford UK

**Keywords:** connectivity map, drug repurposing, MDS, splicing factor mutations

## Abstract

We sought to identify new candidate drugs for repurposing to myelodysplastic syndromes (MDS). Connectivity map analysis was performed on gene expression signatures generated from bone marrow CD34^+^ cells of splicing factor mutant MDS patients. Celastrol and Withaferin A (WA), two top‐ranking compounds identified, markedly inhibited proliferation, arrested the cell cycle and induced apoptosis in leukaemia cells. These compounds also inhibited the viability of primary bone marrow MDS cells. We showed that Celastrol and WA inhibit interleukin‐1 receptor‐associated kinase 4‐mediated nuclear factor kappa‐light‐chain‐enhancer of activated B cells signalling activation in splicing factor mutant MDS and leukaemia cells. Celastrol and WA may represent novel candidate drugs for the treatment of MDS.

## INTRODUCTION

The myelodysplastic syndromes (MDS) represent a heterogeneous group of clonal haematopoietic stem cell malignancies that are characterized by ineffective haematopoiesis leading to peripheral blood cytopenias in one or more lineages.^1^ Approximately 30%–40% of MDS patients develop acute myeloid leukaemia (AML) during the course of their disease. There are few effective treatments for MDS, and allogeneic haematopoietic stem cell (HSC) transplantation is the only potentially curative treatment, but it is only indicated in a small number of cases.^2^ Mutations in RNA splicing regulators (e.g. *SRSF2*, *U2AF1*, *SF3B1* and *ZRSR2*) have been shown to be the most common genomic lesions found in MDS. Emerging data have suggested that strategies to inhibit or modulate spliceosomal genes may have therapeutic value in the treatment of spliceosome‐mutant disorders.^3^ In this study, we used RNA‐seq data that we obtained from bone marrow CD34^+^ cells of MDS patients with splicing factor mutations^4^ to identify differentially expressed genes (DEG) and key gene signatures. These signatures were analysed using the Connectivity Map (CMap) platform, a bioinformatics tool that links drugs, genes and diseases based on gene expression profiles.^5^, ^6^ This approach has been effective in identifying potential therapeutic agents in several diseases, including cancers such as gastric cancer and lung cancer.^7^, ^8^ CMap analysis allowed us to identify compounds that could potentially reverse MDS‐associated gene expression changes and could be repurposed to MDS.

## MATERIALS AND METHODS

See Supporting Information.

## RESULTS AND DISCUSSION

**FIGURE 1:**
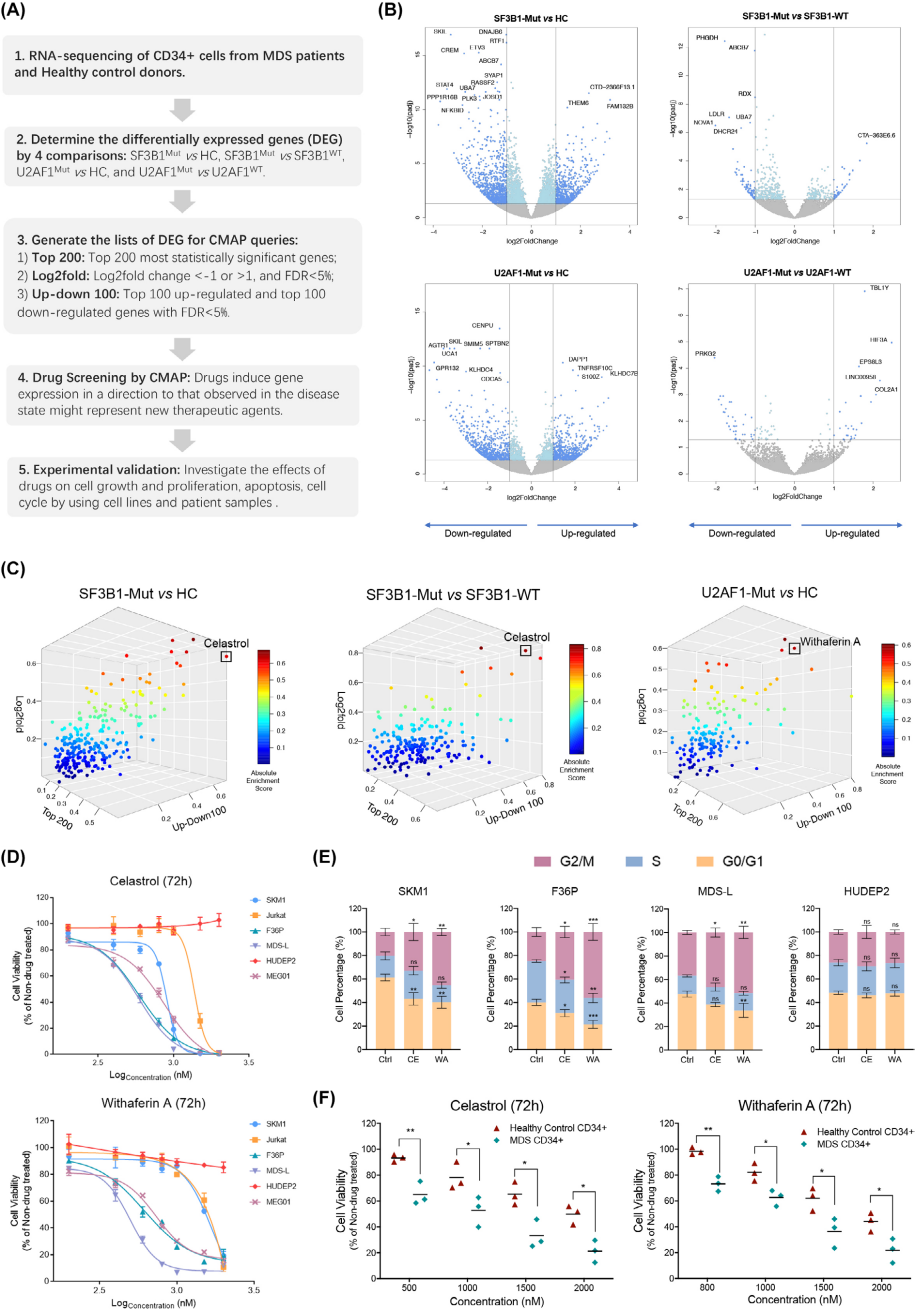
(A) Workflow of GEP‐based drug screening through connectivity mapping. (B) Volcano plots showing the differentially expressed genes in MDS with splicing factor gene mutations. RNA‐Seq data of MDS patients were analysed by DEseq2 package for four different comparisons: *SF3B1* ^Mut^ versus HC, *SF3B1* ^Mut^ versus *SF3B1* ^WT^, *U2AF1* ^Mut^ versus HC, *U2AF1* ^Mut^ versus *U2AF1* ^WT^. Each dot represents a microarray probe annotation matched gene, and some gene symbols were labelled as examples. Vertical lines represent the cut‐off of log_2_fold change corresponding to 1 and −1, and the horizontal indicates an FDR equal to 0.05. (C) Celastrol and Withaferin A were identified as top‐ranking candidates by CMap analysis. The 3‐D scatter plots show the overlapping compounds that were identified in three different CMap queries. Each data point represents a compound. Blue to red colour represents low‐ to high‐absolute enrichment score. Celastrol was identified as one of the top‐ranking candidates in the *SF3B1* ^Mut^ versus HC and in *SF3B1* ^Mut^ versus *SF3B1* ^WT^ comparisons. WA was identified as one of the top‐ranking candidates in the *U2AF1* ^Mut^ versus HC comparison. (D) Drug dose–response curves in cell lines. Viability of cell lines was measured after 72 h Celastrol or WA treatment by adenosine triphosphate (ATP)‐based assay normalized to vehicle‐treated control. Drug doses are represented as logarithm base 10 of nanomolarity (*n* = 3). (E) Cell cycle analysis by propidium iodide (PI) staining. Cell lines were treated with Celastrol (500 nM) and WA (1000 nM) for 12 h, and the cells were stained by PI and analysed by flow cytometry. (F) Cell viability of CD34^+^ cells from MDS patients and healthy donors after 72 h Celastrol or WA treatment with indicated concentrations. All values are plotted as mean ± SD. *p*‐Values were obtained using multiple *t*‐test. *p < 0.05; **p < 0.01; ***p < 0.001. FDR, false discovery rate; GEB, gene expression profiles; MDS, myelodysplastic syndromes; ns, not significant; WA, withaferin A.

First, to determine the DEG in MDS cases with mutations of *SF3B1* and *U2AF1*, we compared the RNA‐Seq data on bone marrow CD34^+^ cells obtained from MDS patients and healthy controls (Figure S1). We compared the following subsets to generate lists of DEG for CMap analysis: *SF3B1* mutant MDS versus healthy controls (*SF3B1* ^Mut^ vs. HC), *SF3B1* mutant MDS versus *SF3B1* wild‐type MDS (*SF3B1* ^Mut^ vs. *SF3B1* ^WT^), *U2AF1* mutant MDS versus healthy controls (*U2AF1* ^Mut^ vs. HC) and *U2AF1* mutant MDS versus *U2AF1* wild‐type MDS (*U2AF1* ^Mut^ vs. *U2AF1* ^WT^). Ranking genes by expression level changes and statistical significance (false discovery rate [FDR]), three lists of DEG for each comparison were generated^9^—(1) Top 200: Top 200 most significant genes, regardless of up‐ or downregulation; (2) Log_2_fold: Genes with a Log_2_fold change lower than −1 or higher than 1, and an FDR <5%; (3) Up‐down 100: Top 100 upregulated and top 100 downregulated genes with an FDR <5% (Figure 1A,B). The DEG lists subsequently served as input to the CMap analysis, except the list of *U2AF1* ^Mut^ versus *U2AF1* ^WT^ that yielded a low number of DEG.

In the *SF3B1* ^Mut^ versus *SF3B1* ^WT^ and *SF3B1* ^Mut^ versus HC comparisons, of 262 overlapped compounds that were identified, Celastrol showed the highest absolute enrichment score and was selected for experimental validation (Figure 1C). Similarly, CMap scored Withaferin A (WA) as one of the top‐ranking candidates in the *U2AF1* ^Mut^ versus HC comparison (Figure 1C). The chemical structures of Celastrol and WA are shown in Figure S2. Celastrol is a pentacyclic triterpene extracted from the *Tripterygium wilfordii* plant; WA is a steroidal lactone isolated from the medicinal plant *Withania somnifera*. Emerging in vitro and in vivo studies have indicated the anti‐cancer effects of Celastrol and WA.^9^, ^10^, ^11^ The detailed drug lists with connectivity scores are shown in Table S1.

Next, we assessed the effects of Celastrol and WA on cell viability in leukaemia cell lines, including MDS‐L, SKM1, F36P, Jurkat and MEG‐01 (Table S2). HUDEP2 cells were used as a non‐leukaemia control cell line. Both Celastrol and WA showed higher cytotoxicity against leukaemic cell lines in a dose‐dependent manner, compared to HUDEP2 cells (Figure 1D). Then, we conducted cell cycle analysis and apoptosis analysis in cell lines. Celastrol or WA induced a significant cell cycle arrest at the G2/M phase in leukaemia cell lines, but not significant in HUDEP2 cells after 12 h drug treatments (Figure 1E). The apoptosis analysis showed an increased apoptotic cell population after 24 h drug treatment in F36P, MDS‐L and SKM1 cell lines. In contrast, Celastrol and WA induced apoptosis in HUDEP2 cells to a much lesser extent (Figure S2).

We tested the effects of Celastrol and WA on CD34^+^ cells isolated from the bone marrow of MDS patients with excess blasts (RAEB2 subtype) and healthy individuals. After 72 h treatment, both drugs showed inhibition of cell viability in a dose‐dependent manner. Notably, these compounds showed significantly higher inhibition of CD34^+^ cells from MDS patients compared to CD34^+^ cells from healthy controls (Figure 1F). Overall, these results indicate that primary MDS cells are preferentially sensitive to Celastrol and WA.

We then investigated the effects of Celastrol or WA in combination with an existing compound used for the treatment of MDS. In drug combination assays, the cytotoxic effects of Celastrol or WA on leukaemia cells were enhanced by azacitidine (Figure S3). The synergy between compounds was measured by the highest single agent (HSA) model using SynergyFinder, where the synergy score (*δ*‐score) quantifies the excess over the highest single drug response. A positive *δ*‐score suggests synergistic effects. The *δ*‐score of the Celastrol and Azacitidine combination is 12.68 in SKM1 cells and 12.602 in MDS‐L cells, whereas the WA and azacitidine combination treatment exhibited a higher *δ*‐score with 22.456 in SKM1 cells and 16.692 in MDS‐L cells (Figure S3). Interestingly, combination treatment using Celastrol and WA showed a strong synergistic effect in SKM‐1 cells (*δ*‐score = 9.742) and MDS‐L cells (*δ*‐score = 4.438), higher than in HUDEP2 cells (*δ*‐score = 1.668) (Figure S4). These data suggest that the use of Celastrol or WA in combination with Azacitidine may have greater efficacy in inhibiting leukaemic cell growth.

**FIGURE 2:**
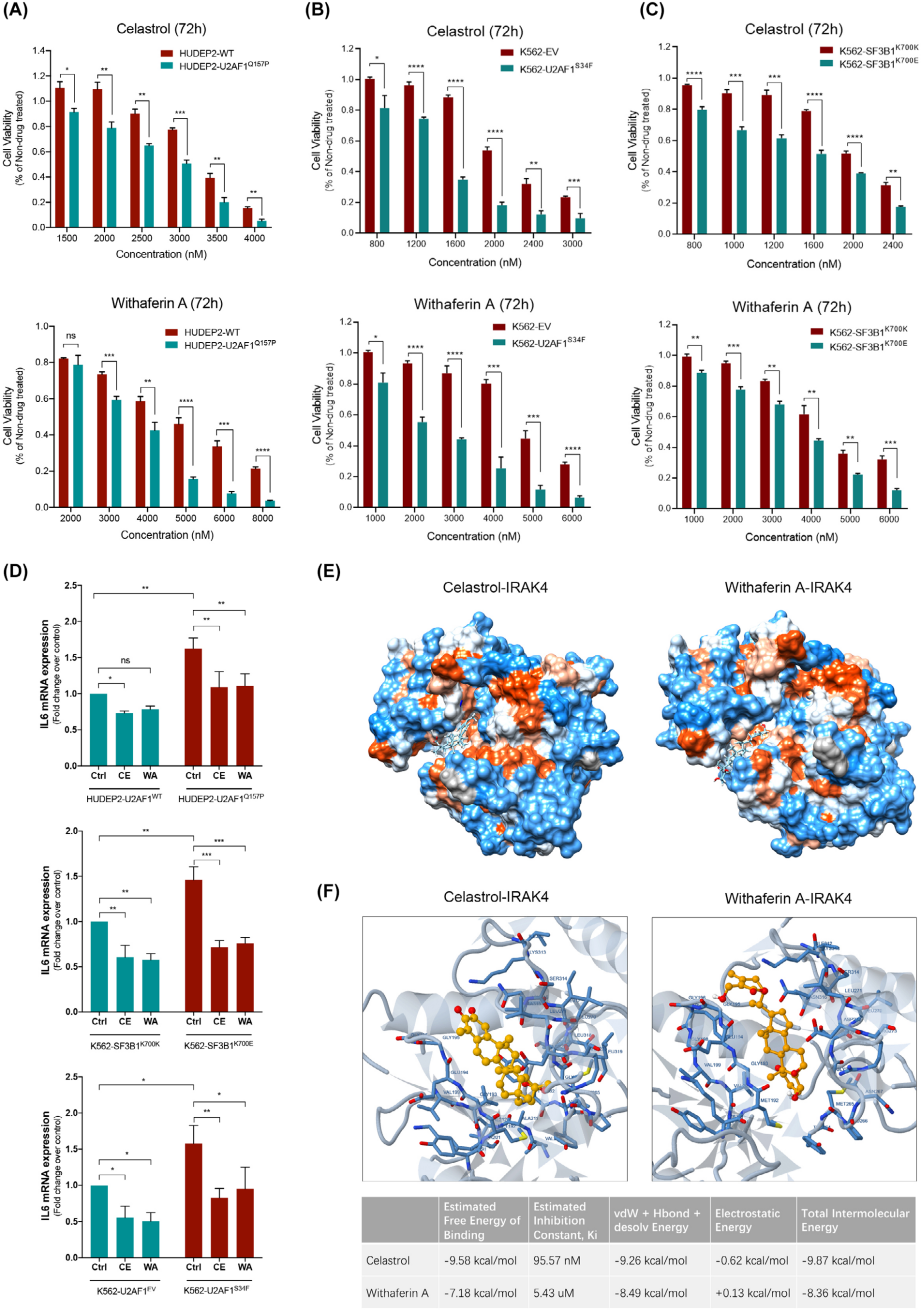
(A) Viability of HUDEP2‐U2AF1^WT^ and HUDEP2‐U2AF1^Q157P^ after 72 h Celastrol or WA treatment with indicated concentrations. Since HUDEP2 cells were less sensitive than MDS/AML cell lines to Celastrol and WA treatment, we used a higher dose range to test the cell viability after 72 h (Celastrol: 1.5–4 μM; WA: 2–8 μM). (B) Viability of K562‐EV and K562‐U2AF1^S34F^ after 72 h Celastrol or WA treatment with indicated concentrations. (C) Viability of K562‐SF3B1^K700K^ and K562‐SF3B1^K700E^ after 72 h Celastrol or WA treatment with indicated concentrations. (D) IL‐6 mRNA levels in spliceosome mutant cells compared to their respective WT counterpart (HUDEP2‐U2AF1^WT^ and HUDEP2‐U2AF1^Q157P^, K562‐EV and K562‐U2AF1^S34F^, K562‐SF3B1^K700K^ and K562‐SF3B1^K700E^) after 4 h Celastrol (1000 nM) or WA (2000 nM) treatment. (E) Molecular docking of Celastrol or WA with IRAK4, simulated by Swissdock. The colours indicate the hydrophobicity of the binding surface, from dodger blue for the most hydrophilic, to white, to orange red for the most hydrophobic. (F) Binding modes and docking calculations of Celastrol or WA interacting with IRAK4 carried out by using DockingServer. The drugs were shown as a stick‐and‐ball model (orange balls are carbon atoms and red balls are oxygen atoms). All values are plotted as mean ± SD. *p*‐Values in (A–C) were obtained using multiple *t*‐test. *p*‐Values in (D) were obtained using two‐way ANOVA with the Tukey's multiple comparisons test. *p < 0.05; **p < 0.01; ***p < 0.001; ****p < 0.0001. AML, acute myeloid leukaemia; ANOVA, analysis of variance; IL, interleukin; MDS, myelodysplastic syndromes; ns, not significant; WA, withaferin A; WT, wild type.

To investigate whether Celastrol and WA preferentially inhibit cell proliferation in association with the presence of splicing factor gene mutations, we tested the cytotoxicity of these drugs in HUDEP2 cells with U2AF1^Q157P^ mutation introduced via CRISPR/Cas9, K562 with SF3B1^K700E^ mutation and K562 cells overexpressing U2AF1^S34F^. HUDEP2‐U2AF1^Q157P^ cells were significantly more sensitive to Celastrol and WA than the U2AF1 wild‐type cells (Figure 2A). K562‐SF3B1^K700E^ cells showed preferential sensitivity to these drugs compared with isogenic K562‐SF3B1^K700K^ cells (Figure 2B). K562 cells overexpressing U2AF1^S34F^ similarly demonstrated higher sensitivity to both drugs compared to empty vector‐transfected K562 cells (Figure 2C). These results indicate that spliceosome mutant cells showed a significantly higher sensitivity to Celastrol and WA treatment compared to wild‐type cells.

Some studies have reported the potential of Celastrol and WA as therapeutic agents against various disorders, including solid cancer, rheumatoid arthritis and obesity,^9^, ^10^, ^11^ but their mode of action remains largely unclear. Interestingly, Celastrol has been shown to suppress the proliferative potential of AML cells by inhibiting the Myb/p300 interaction.^12^ Previous studies have demonstrated that spliceosome gene mutations enhance innate immune signalling, contributing to the pathogenesis of MDS and AML.^13^, ^14^, ^15^ Mutations in SF3B1, U2AF1 and SRSF2 can enhance nuclear factor kappa‐light‐chain‐enhancer of activated B cells (NF‐κB) activity and inflammatory cytokine production (such as interleukin [IL]‐6) in myeloid malignant cells.^13^, ^14^ Therefore, we investigated the effects of Celastrol and WA on IL‐6 expression levels in splicing factor mutant cells. The quantitative polymerase chain reaction results show that the untreated U2AF1^Q157P^ mutant HUDEP2 cells, SF3B1^K700E^ mutant K562 cells and U2AF1^S34F^ overexpressing K562 cells had significantly higher basal levels of IL‐6 mRNA expression, a downstream target gene of NF‐κB signalling, compared to the wild‐type cells. The relationship between compounds identified by our CMap analysis and specific pathways, such as interleukin‐1 receptor‐associated kinase 4 (IRAK)4/NF‐κB signalling, that are involved in MDS pathophysiology and represent therapeutic targets, demonstrates the impact of DEG on therapeutic choices. Celastrol or WA treatment significantly inhibited IL‐6 expression levels in both spliceosome mutant and wild‐type cells (Figure 2D). It would be interesting to evaluate the effects of Celastrol and WA in diseases characterized by overexpression of IL‐6, such as multicentric Castleman disease.^16^

A molecular docking simulation using Swissdock provided a possible mechanism of Celastrol and WA interacting with IRAK4, a critical kinase in regulating and activating NF‐κB signalling in myeloid leukaemia.^13^, ^15^ The docking model showed that the adenosine triphosphate (ATP)‐binding cleft of the IRAK4 kinase domain was occupied by Celastrol or WA, sandwiched between a bilobal structure (Figure 2E). Figure 2F shows the ligand–protein interaction modes and docking calculations carried out by using DockingServer. Celastrol binding to IRAK4 might be mediated by numerous forms of interaction, including a hydrogen bond with MET265, polar interaction with LYS313, ASN316, ALA211 and hydrophobic interaction with LEU318, MET192, VAL200 and ALA315. WA may bind to IRAK4 through interacting with VAL200, SER269, MET192, PHE197, LEU271, ALA315 and ASP272. These amino acids compose the core structure of the ATP‐binding site for the IRAK4 kinase domain.^17^

Our study has some limitations. The CMap platform is based on microarray data, which may not fully capture all transcriptomic changes from RNA‐seq. In addition, genes that did not have a match in the reference microarray platform may contain important biological information. It is recognized that the use of RNA‐seq data may introduce potential biases, including challenges in capturing low‐abundance transcripts and variability across samples. The use of computational models, such as molecular docking, may oversimplify biological interactions and should be interpreted cautiously. While the identified compounds were cross‐checked with known databases, further validation using experimental and computational methods, such as protein–protein interaction (PPI) analyses, would be beneficial to verify connections between DEG and the selected compounds.

In conclusion, our study suggests that Celastrol and WA have high potential to serve as novel candidate drugs for the treatment of MDS. Our data provide evidence of the involvement of splicing modulation in the anti‐proliferative activity of Celastrol and WA in leukaemia cells. Mechanistically, Celastrol and WA may inhibit IRAK4‐mediated NF‐κB signalling activation in splicing factor mutant cells. Further studies, including in vivo studies in patient‐derived xenografts and other animal models, are warranted to unravel the exact mechanisms of these drugs in suppressing leukaemia cell growth.

## AUTHOR CONTRIBUTIONS

TS, SS, JL and HD performed the research; TS, HCW and AP analysed the data; PLC and DRH contributed essential reagents or tools; TS, JB and AP designed the research study; TS, PLC, JB and AP wrote the paper.

## CONFLICT OF INTEREST STATEMENT

No conflicts of interest are declared.

## Supporting information


Data S1.

Table S1.

Table S2.
