# Supplementary material for: Identification of new candidate drugs in myelodysplastic syndromes with splicing factor mutations by transcriptional profiling and connectivity map analysis
Source: Br J Haematol. 2025 Feb 23;206(4):1086–91. doi: 10.1111/bjh.20026 (PMC11985364; doi:10.1111/bjh.20026)
Supplement: Supplementary file 1 — Data S1. [file BJH-206-1086-s001.docx]

**SUPPLEMENTARY MATERIAL**

**Identification of new candidate drugs in myelodysplastic syndromes with splicing factor mutations by transcriptional profiling and connectivity map analysis**

Tianyu Sun^1^, Shalini Singh^1^, Hayson Chenyu Wang^2,3^, Juseong Lee^1^, Hamid Dolatshad^1^, Pak Leng Cheong^4^, Douglas R Higgs^4^, Jacqueline Boultwood^1*^, Andrea Pellagatti^1*^

1. Nuffield Division of Clinical Laboratory Sciences, Radcliffe Department of Medicine, University of Oxford, Oxford, UK

2. Shanghai Ninth People's Hospital, Shanghai Jiaotong University School of Medicine, Shanghai, China

3. Nuffield Department of Surgical Sciences, University of Oxford, Oxford, UK

4. MRC Weatherall Institute of Molecular Medicine, Radcliffe Department of Medicine, University of Oxford, Oxford, UK

Materials and Methods

1. RNA-sequencing data

The RNA-seq dataset on bone marrow CD34^+^ cells from myelodysplastic syndrome (MDS) patients was obtained from a previous study published by our group (1). Data from a total of 73 MDS patients and 8 healthy controls were included in this current study. Of the 73 MDS cases, 28 were SF3B1 mutant, 6 U2AF1 mutant, and 39 had no mutations in the splicing factor genes *SF3B1*, *SRSF2*, *U2AF1* and *ZRSR2*.

2. Identification of differentially expressed genes

Differentially expressed gens (DEG) are genes that show significant differences in expression levels between two or more groups**.** Differential gene expression analyses were performed using R/Bioconductor package DESeq2 (http://www.bioconductor.org/). DESeq2 provides statistical routines for determining differential expression in digital gene expression data using a model based on the negative binomial distribution. The resulting P-values were adjusted using the Benjamini and Hochberg’s approach for controlling the false discovery rate (FDR) (2). The significance of the gene expression difference was determined with an adjusted P-value (padj) <0.05 found by DESeq2. To ensure rigorous and biologically meaningful results, we applied the following parameters in our analysis: 1) Cutoff Parameters: Genes with an adjusted P-value (padj) <0.05 and a log2 fold change (LFC) >1 or <-1 were considered significantly and biologically differentially expressed. These thresholds balance statistical significance with biological relevance. 2) Normalization: The median-of-ratios normalization method, provided by DESeq2, was utilized to account for differences in library size and composition across samples, ensuring accurate and consistent comparison of gene expression levels. We acknowledge potential biases in low-expression genes, which may be more susceptible to variability and noise. While DESeq2’s empirical Bayes shrinkage approach helps mitigate these issues, such results were interpreted cautiously to avoid overestimation of differential expression. Additionally, RNA-seq is inherently limited in detecting low-abundance transcripts and may introduce amplification biases during library preparation. These factors should be considered when interpreting the results, particularly for low-expression genes.

3. Identification of candidate drugs using connectivity map

The connectivity map (CMap) is a database with a collection of gene expression data sets from cultured human cell lines treated with various small molecules (3, 4). Since CMap is a microarray-based platform, gene symbols of the DEG obtained from the analyses of the RNA-seq data were converted to microarray probe annotations. The final lists of DEG that were used for a query in CMap excluded non-matching genes. Genes that did not have a direct match in the database were excluded from the analysis, as they could not be mapped to the reference microarray platform. We acknowledge that this exclusion may influence the results, particularly if unmapped genes are biologically significant. To mitigate this, we ensured that the majority of key genes identified in our differential expression analysis were successfully mapped. Additionally, we cross-checked the unmapped genes for potential enrichment in pathways or functions to confirm that their exclusion did not introduce significant biases. CMap utilizes a gene-set enrichment metric (Kolmogorov-Smirnov) statistic to rank order individual treatment instances by their similarity to a given query signature. The output drugs given by CMap were ranked by a ‘connectivity score’ normalized from −1 to +1 to reflect the connection between the expression profiles. A positive connectivity score suggesting the drug treatment can mimic the expression pattern of a disease state. In contrast, a negative connectivity score stands for a reversed transcriptomic mapping of disease state to drug treatment. Here, only those compounds with a negative connectivity score that were identified by the analysis of all three gene lists were considered of interests. These drugs were ranked by the absolute value of connectivity score in descending order, and only top-scoring drugs were used for the selection of candidates for wet-lab experiments. In light of existing data concerning chemotherapy drugs, epigenetic modulators, and other drugs used for MDS treatment, compounds without validated mechanisms that modulate dysregulated pathways in haematological malignancies were prioritised for experimental validation.

**4. Cell lines**

HUDEP2 cells with heterozygous U2AF1 Q157P mutation were generated thorough plasmid-based methodology, using CRISPR/Cas9 system combined with single-stranded oligodeoxynucleotide (ssODN) donors, as described previously (5). We first created an intermediate clone by targeting U2AF1 at the region surrounding codon Q157 (sgRNA sequences: U2AF1-sgRNA-TOP caccGCACCCCATCTCATACTGA; U2AF1-sgRNA-BOTTOM aaacTCAGTATGAGATGGGGTGG). A ssODN was included with the intention to introduce synonymous variant in one allele to prevent re-editing (U2AF1-ssODN-WT: CACGTCACTGGCCACTCCTCACTCACCCCATCTCgTACTGtCGaCAGCAGGCTTCTC

TGAAGTCCGTCACGGGTGACAGCTCGGCGTGGATCGGC; small letters depict deliberate synonymous variants). After the first step, an intermediate clone with a heterozygous 5bp deletion (NG_029455.1:g.17917_17922delAGATG) surrounding codon Q157 was created. The other allele was unmodified despite inclusion of the ssODN intended to protect a wildtype allele. In the second step, a sgRNA specifically targeting the 5bp deletion allele was used to cut only the mutated allele (U2AF1-5bpdel-TOP: caccGCCTGCTGCCGTCAGTAT; U2AF1-5bpdel-BOTTOM: aaacATACTGACGGCAGCAGGC). Another ssODN was added to 1. introduce the Q157P mutation, and 2. introduce deliberate synonymous variants nearby to prevent re-editing of the mutated allele (U2_Q157P_ssODN5: CACGTCACTGGCCACTCCTCACTCACCCCATCTCATACgGcCtGCAGCAGGCTTCTCTGAAGTCCGTCACGGGTGACAGCTCGGCGTGGATCGGC; small letters depict the Q157P mutation and deliberate synonymous variants). After a second round of editing, the final clone contains an allele with Q157P mutation and two synonymous variants.

K562 cells overexpressing U2AF1S34F were generated by using retroviral pGCDNsam-IRES-EGFP plasmids containing U2AF1S34F cDNA. Vector plasmids were co-transfected with Gag/Pol plasmids and vesicular stomatitis virus glycoprotein envelope using jetPRIME transfection reagent (Polyplus transfection) into HEK293T cells as described previously to obtain high-titer retrovirus stock (6). Retroviruses were harvested after 48h and 72h of transfection. Harvested retroviruses were concentrated by ultracentrifugation (Beckman Coulter, Brea, CA, USA; Ultracentrifuge Rotor SW28) at 28000 rpm for 3h at 4°C. Spinoculation of K562 cells was performed in the presence of 8 μg/ml polybrene (Sigma-Aldrich) at 800 ×g, 32°C for 2 hours. The transduced K562 cells were selected after overnight incubation with retroviruses using FACS sorting for GFP positive cells.

K562 cells with the SF3B1K700E mutation and isogenic SF3B1K700K K562 cells were purchased from Horizon Discovery.

**5. Patient samples and isolation of CD34^+^ cells from bone marrow mononuclear cells**

Bone marrow samples from MDS patients were obtained with approval by the relevant research ethics committee (reference 17/YH/0322) and with written informed consent by the patients. Bone marrow CD34^+^ cells from healthy individuals were purchased from LONZA. Frozen bone marrow mononuclear cells from MDS patients were thawed and treated with DNase 1 (100U/ml) (ThermoFisher Scientific) in Dulbecco's PBS (pH 7.0-7.2) (Sigma Aldrich), containing 0.5 mM MgCl_2_, 1 mM CaCl_2_ and 0.5% BSA (Sigma Aldrich) for 45 mins at room temperature. CD34^+^ cells were isolated using the CD34 MicroBead Kit UltraPure (Miltenyi Biotec), according to the manufacturer’s protocol. The purity of the isolated CD34^+^ cells was measured by flow cytometry (using CD34-PE, human, Clone: AC136, Miltenyi Biotec) and was >85% for all samples tested.

6. Compounds and cell culture

Celastrol (Stratech Scientific), Withaferin A (WA) (Fisher Scientific), and Azacitidine (Sigma-Aldrich) were utilized as experimental drugs. The drugs were dissolved in dimethylsulphoxide (DMSO) and stored at -20°C. All leukaemia cell lines were maintained in RPMI 1640 (Sigma-Aldrich) supplemented with 10% fetal bovine serum (20% in SKM-1 cell line), and 1% Penicillin-Streptomycin-L-Glutamine (PSG; Sigma-Aldrich). SKM1, F36P and MDS-L cell line was further supplemented with GM-CSF or IL-3 (1 ng/ml GM-CSF in SKM1, 10 ng/ml IL-3 in MDS-L, and 1 ng/ml IL-3 in F36P, Miltenyi Biotec). HUDEP2 cell line were grown in StemSpan™ serum-free hematopoietic cell expansion media (Stemcell technologies) supplemented with 1% PSG, 50 ng/ml SCF (Stemcell technologies), 3 IU/ml EPO (Stemcell technologies), 1 uM Dexamethasone (Stemcell technologies) and 1 ug/ml Doxycycline (Stemcell technologies). Human bone marrow CD34^+^ cells were cultured in StemSpan Serum-Free Expansion Medium (Stemcell Technologies) containing StemSpan CD34^+^ Expansion Supplement (Stemcell Technologies). All cells were grown in an incubator at 37°C and 5% CO_2_.

7. Cell viability assay

Cells were plated in 96-well plates and treated with a range of concentrations for each candidate drug, or with vehicle (DMSO) as control, for 72 hours. The cell viability was analysed by using CellTiter-Glo® Luminescent Cell Viability Assay Kit (Promega) and SpectraMax i3x plate reader (Molecular Devices) according to manufacturer’s protocol. The nonlinear regression curves were drawn using GraphPad Prism. Percentages of viable cells were calculated by normalizing to the DMSO controls. Synergy score was generated using SynergyFinder (https://synergyfinder.fimm.fi) using the HSA model.

8. Cell cycle analysis

Cells were seeded in 6 well plates and exposed to Celastrol (300 nM) or WA (1000 nM) for 48hours. Fixation and permeabilization of cells were performed by dropwise addition of 70% ethanol while vortexing and incubation on ice for at least 1 hour. Permeabilized cells were washed with PBS and treated with 0.04mg/ml propidium iodide (Biolegend) and 0.1 mg/ml RNaseA (Sigma-Aldrich) for 30 minutes at room temperature in the dark. The DNA content was analysed via flow cytometer (BD LSR II, BD Biosciences). The cell cycle was analysed using FlowJo software.

9. Cell Apoptosis analysis

Cells were seeded in 6 well plates and treated with Celastrol (500 nM) and WA (1500 nM) for 24 hours. The apoptotic cells were determined by staining with Annexin V-FITC and propidium iodide (Biolegend) according to manufacturer’s protocol. The cells were analysed on a flow cytometer (BD LSR II, BD Biosciences). The population of apoptotic cells was analysed using FlowJo software.

**10. Quantitative Real Time-PCR**

K562 cells (5×10^5^ cells/mL) were treated with Celastrol or WA for 4 h. Total RNA was isolated using the TRIzol reagent (Life Technologies), and cDNA was synthesized using the High-Capacity cDNA Reverse Transcription Kit (Life Technologies), according to the manufacturer's instructions. Quantitative reverse transcriptase (RT)-PCR was performed using LightCycler480 SYBR Green Master Mix (Roche) and the LightCycler 96 Instrument (Roche). Primer sequences used in this study were designed as the following: IL6, forward, 5′- AGACAGCCACTCACCTCTTCAG-3′, reverse, 5′- TTCTGCCAGTGCCTCTTTGCTG-3′. B2M was used as an internal standard. All reactions were run in triplicate, and data were analyzed using the 2^−ΔΔCT^ method (7).

**11. Molecular docking**

Molecular Docking studies were performed in SwissDock (https://www.swissdock.ch) and DockingServer (http://www.dockingserver.com). The crystal structure of IRAK4 was obtained from the Protein Data Bank (PDB) with the accession code 2NRU (8). Docking step was firstly performed using SwissDock web service based on the docking software EADock DSS. The ligand and receptor files in the appropriate formats were uploaded to the web-based server for docking study. A grid was designed in which many binding modes were generated for the most favourable bindings. Simultaneously, their CHARMM energies are estimated on the grid. The binding modes with the most favourable energies were evaluated with Fast analytical continuum treatment of solvation (FACTS), and clustered. Binding modes were scored using their FullFitness and clustered. Clusters were then ranked according to the average FullFitness of their elements. Results of the SwissDock were visualized by UCSF Chimera package (9). Docking calculations and ligand-protein interaction modes were carried out using DockingServer (10). The MMFF94 force field was used for energy minimization of ligand molecule. Gasteiger partial charges were added to the ligand atoms. Non-polar hydrogen atoms were merged, and rotatable bonds were defined. Docking calculations were carried out on IRAK4 protein model. Essential hydrogen atoms, Kollman united atom type charges, and solvation parameters were added with the aid of AutoDock tools. Affinity (grid) maps of 26×28×28 Å grid points and 0.375 Å spacing were generated using the Autogrid program. AutoDock parameter set- and distance-dependent dielectric functions were used in the calculation of the van der Waals and the electrostatic terms, respectively. Docking simulations were performed using the Lamarckian genetic algorithm (LGA) and the Solis & Wets local search method. Initial position, orientation, and torsions of the ligand molecules were set randomly. Each docking experiment was derived from 2 different runs that were set to terminate after a maximum of 250000 energy evaluations. The population size was set to 150. During the search, a translational step of 0.2 Å, and quaternion and torsion steps of 5 were applied.

**12. Statistics**

Statistical analysis was performed using GraphPad Prism 10 (San Diego, California, USA). A P-value <0.05 was considered significant. The type of statistical test used and the results including P values, means and standard error of the mean are shown in the figures and figure legends.

**Supplementary references**

1. Pellagatti A, Armstrong RN, Steeples V, Sharma E, Repapi E, Singh S, et al. Impact of spliceosome mutations on RNA splicing in myelodysplasia: dysregulated genes/pathways and clinical associations. Blood. 2018;132(12):1225-40.

2. Love MI, Huber W, Anders S. Moderated estimation of fold change and dispersion for RNA-seq data with DESeq2. Genome Biol. 2014;15(12):550.

3. Lamb J, Crawford ED, Peck D, Modell JW, Blat IC, Wrobel MJ, et al. The Connectivity Map: using gene-expression signatures to connect small molecules, genes, and disease. Science. 2006;313(5795):1929-35.

4. Keenan AB, Wojciechowicz ML, Wang ZC, Jagodnik KM, Jenkins SL, Lachmann A, et al. Connectivity Mapping: Methods and Applications. Annu Rev Biomed Da S. 2019;2:69-92.

5. Moir-Meyer G, Cheong PL, Olijnik AA, Brown J, Knight S, King A, et al. Robust CRISPR/Cas9 Genome Editing of the HUDEP-2 Erythroid Precursor Line Using Plasmids and Single-Stranded Oligonucleotide Donors. Methods Protoc. 2018;1(3).

6. Singh S, Ahmed D, Dolatshad H, Tatwavedi D, Schulze U, Sanchi A, et al. SF3B1 mutations induce R-loop accumulation and DNA damage in MDS and leukemia cells with therapeutic implications. Leukemia. 2020.

7. Livak KJ, Schmittgen TD. Analysis of relative gene expression data using real-time quantitative PCR and the 2(-Delta Delta C(T)) Method. Methods. 2001;25(4):402-8.

8. Wang Z, Liu J, Sudom A, Ayres M, Li S, Wesche H, et al. Crystal structures of IRAK-4 kinase in complex with inhibitors: a serine/threonine kinase with tyrosine as a gatekeeper. Structure. 2006;14(12):1835-44.

9. Grosdidier A, Zoete V, Michielin O. SwissDock, a protein-small molecule docking web service based on EADock DSS. Nucleic Acids Res. 2011;39(Web Server issue):W270-7.

10. Bikadi Z, Hazai E. Application of the PM6 semi-empirical method to modeling proteins enhances docking accuracy of AutoDock. J Cheminform. 2009;1:15.

**
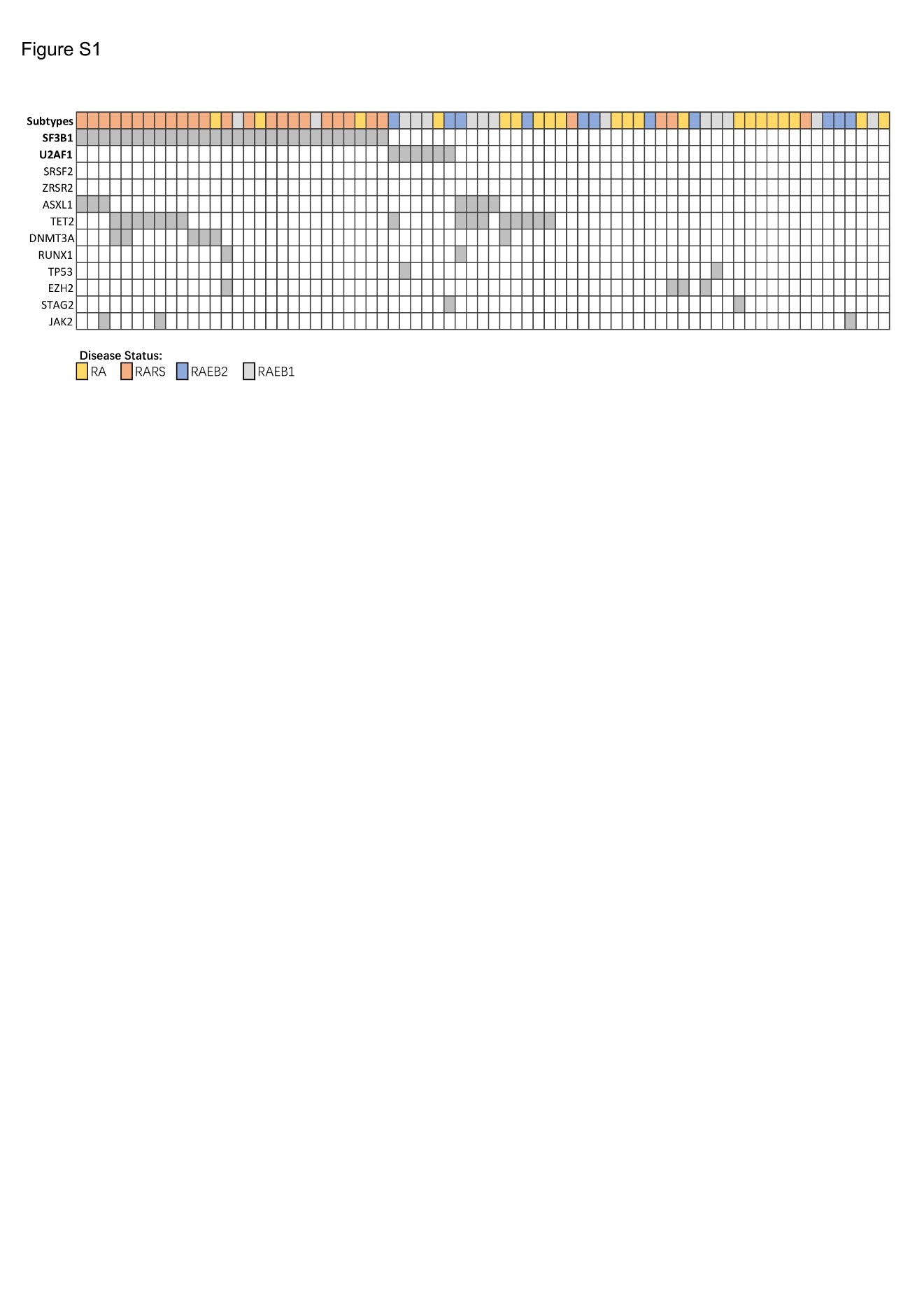
**

**Figure S1.** Disease subtypes and mutations status of MDS patients. MDS patients were diagnosed as refractory anemia (RA), RA with ring sideroblasts (RARS), RA with excess blasts (RAEB1 and RAEB-2) according to 2008 World Health Organization (WHO) classification system. Splicing factor gene mutations and other common gene mutations were detected by targeted next-generation sequencing (1).


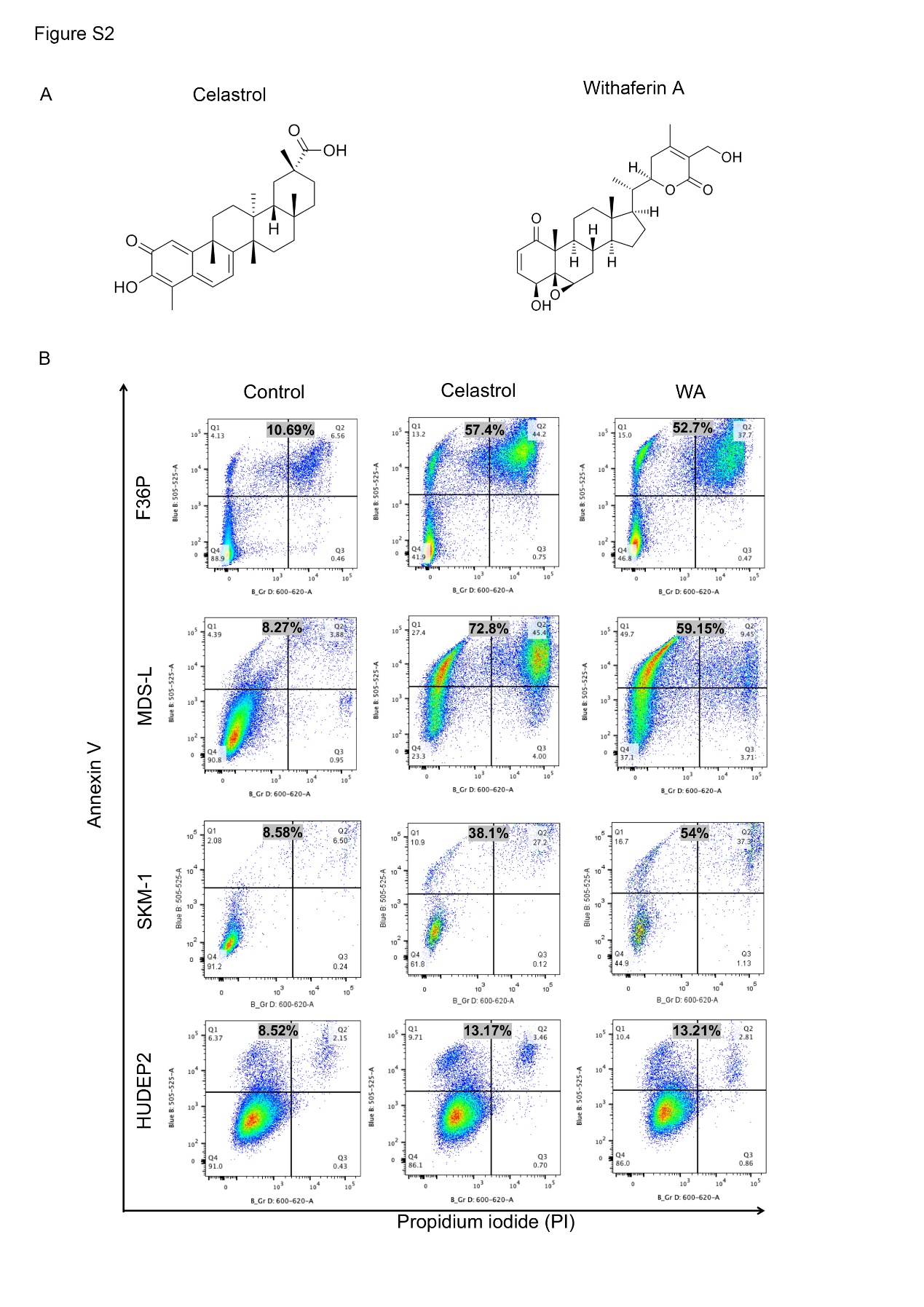


**Figure S2.** (A) Chemical structure of Celastrol and WA. (B) Cell apoptosis induced by Celastrol and WA. F36P, SMK1, MDS-L and HUDEP2 cells were treated with 500 nM Celastrol or 1000 nM WA for 24 h. Cell apoptosis was measured by using Annexin-V/PI dual staining. A single-positive staining for Annexin-V is an early indicator of apoptosis (Annexin-V+/PI-, upper-left area), and the double-positive staining for Annexin and PI indicates late apoptosis or necrosis (Annexin-V+/PI+, upper-right area).


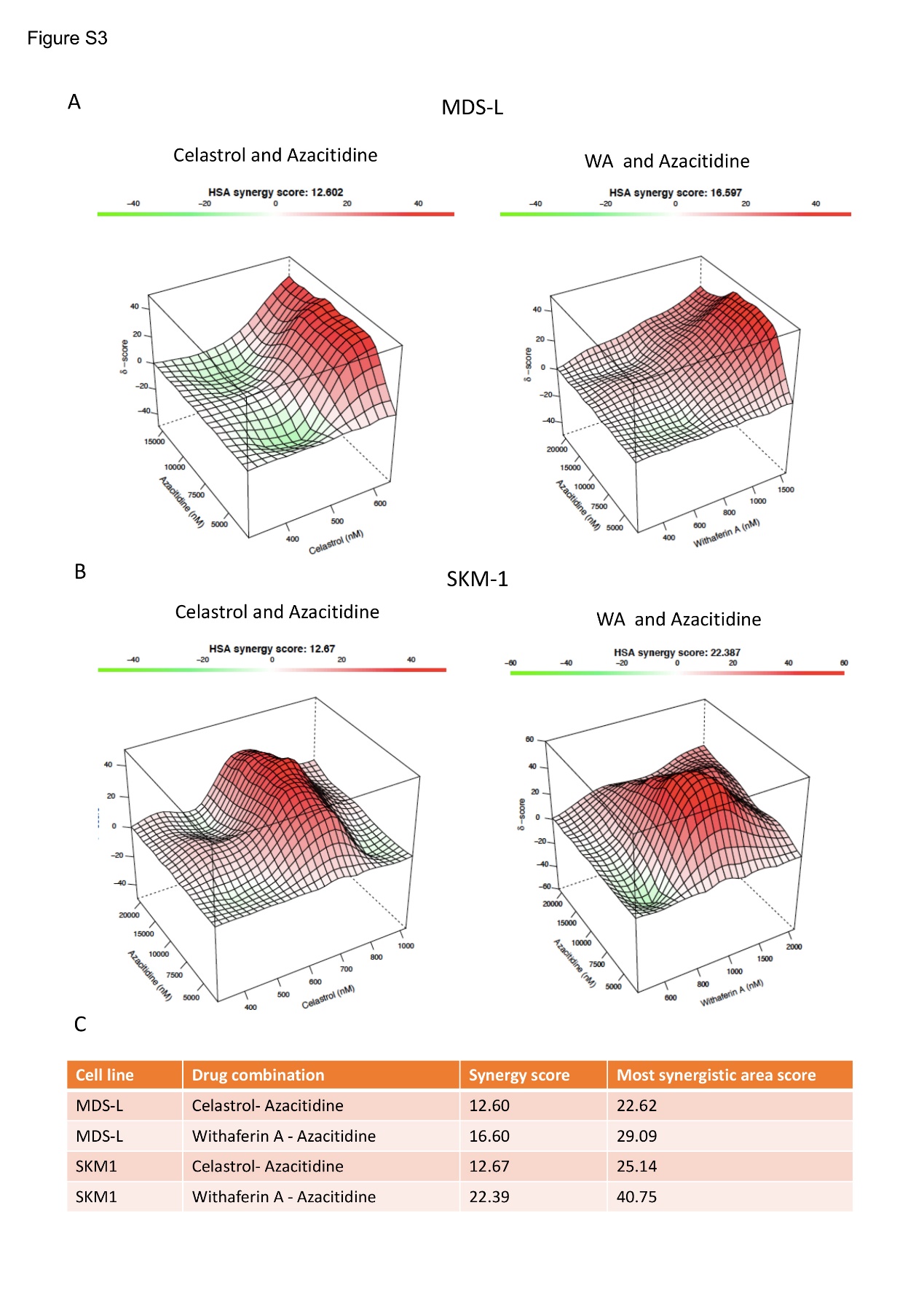


**Figure S3.** Visualization of 3D synergy maps showing overall synergy scores (δ-score) for combination treatment of Celastrol or WA with Azacitidine on (A) MDS-L cells and (B) SKM-1 cells. A positive synergy score (δ-score) (Red) denotes synergy. (C) Table showing the synergy score and most synergistic area score of drug combinations in SKM-1 and MDS-L cells.


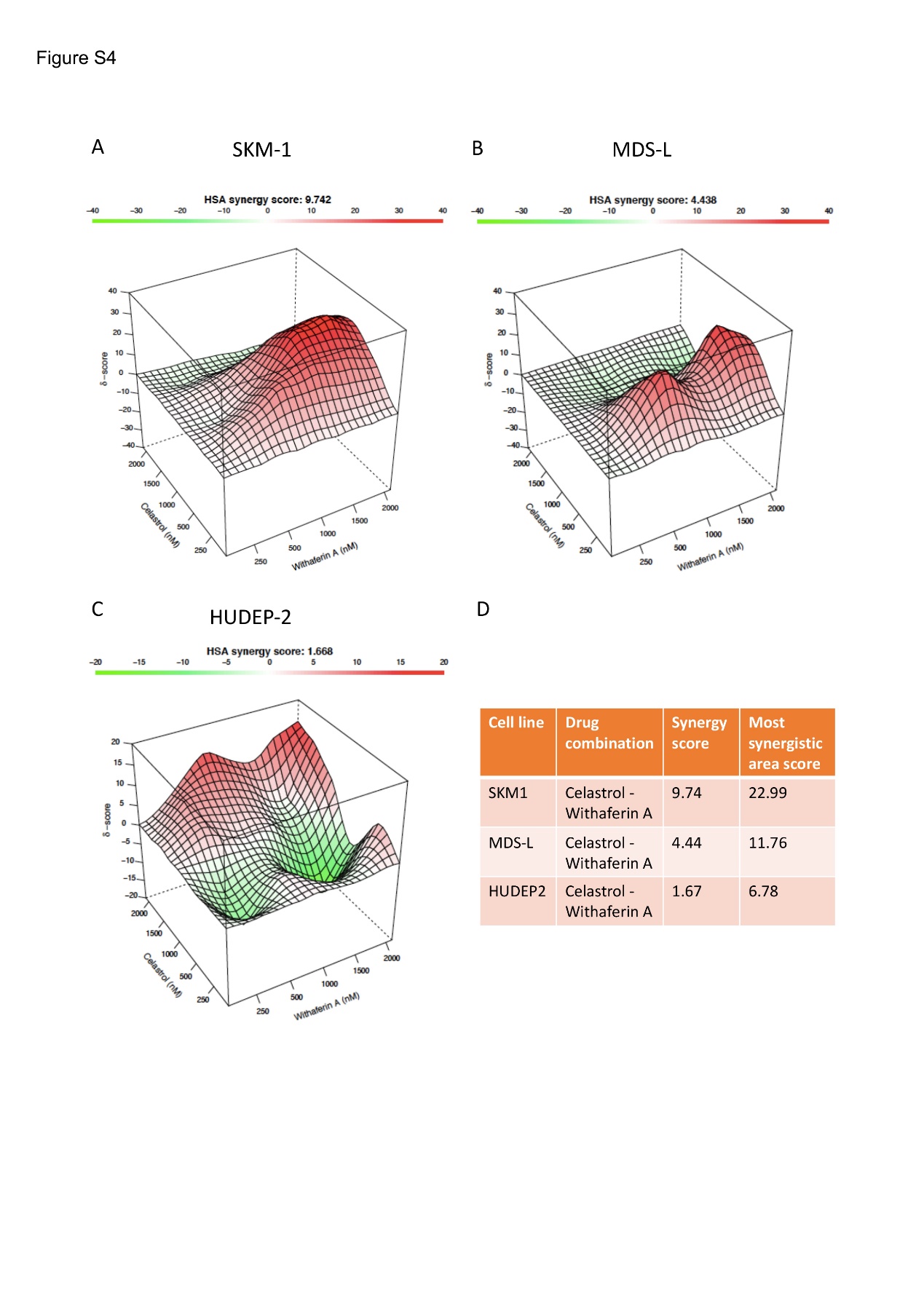


**Figure S4.** Visualization of 3D synergy maps showing overall synergy scores (δ-score) for combination treatment of Celastrol and WA on (A) SKM1 cells, (B) MDS-L cells, and (C) HUDEP-2 cells. A positive synergy score (δ-score) (Red) denotes synergy. (D) Table showing the synergy score and most synergistic area score of drug combinations in SKM-1, MDS-L, and HUDEP2 cells.
